# Risk factors associated with 28-day all-cause mortality in older severe COVID-19 patients in Wuhan, China: a retrospective observational study

**DOI:** 10.1038/s41598-020-79508-3

**Published:** 2020-12-22

**Authors:** Yi Jiang, Subi Abudurexiti, Meng-Meng An, Da Cao, Jie Wei, Ping Gong

**Affiliations:** 1 Department of Emergency, First Affiliated Hospital of Dalian Medical University, 222 Zhongshan Road, Xigang District, Dalian, 116011 Liaoning China; 2 Department of Emergency, General Hospital of Tianjin Medical University, Tianjin, 300052 China; 3 Intensive Care Unit, Affiliated Dalian Friendship Hospital of Dalian Medical University, Dalian, 116011 Liaoning China; 4 Department of Emergency, Southeast University Zhongda Hospital, Nanjing, 210009 Jiangsu China; 5 Department of Emergency, Renmin Hospital of Wuhan University, 238 Jiefang Road, Wuchang District, Wuhan, 430060 Hubei China

**Keywords:** Medical research, Risk factors

## Abstract

We aimed to analyse clinical characteristics and identify risk factors predicting all-cause mortality in older patients with severe coronavirus disease 2019 (COVID-19). A total of 281 older patients with severe COVID-19 were categorized into two age groups (60–79 years and ≥ 80 years). Epidemiological, clinical, and laboratory data, and outcome were obtained. Patients aged ≥ 80 years had higher mortality (63.6%) than those aged 60–79 years (33.5%). Anorexia and comorbidities including hypertension, diabetes and COPD, higher levels of lactate dehydrogenase (LDH), osmotic pressure, C-reactive protein, D-dimer, high-sensitivity troponin I and procalcitonin, and higher SOFA scores were more common in patients aged > 80 years than those aged 60–79 years and also more common and higher in non-survivors than survivors. LDH, osmotic pressure, C-reactive protein, D-dimer, high-sensitivity troponin I, and procalcitonin were positively correlated with age and sequential organ failure assessment (SOFA), whereas CD8+ and lymphocyte counts were negatively correlated with age and SOFA. Anorexia, comorbidities including hypertension, diabetes, and chronic obstructive pulmonary disease (COPD), LDH, osmotic pressure, and SOFA were significantly associated with 28-day all-cause mortality. LDH, osmotic pressure and SOFA were valuable for predicting 28-day all-cause mortality, whereas the area under the receiver operating characteristic curve of LDH was the largest, with sensitivity of 86.0% and specificity of 80.8%. Therefore, patients with severe COVID-19 aged ≥ 80 years had worse condition and higher mortality than did those aged 60–79 years, and anorexia and comorbidities including hypertension, diabetes, COPD, elevated plasma osmotic pressure, LDH, and high SOFA were independent risk factors associated with 28-day all-cause mortality in older patients with severe COVID-19. LDH may have the highest predictive value for 28-day all-cause mortality in all examined factors.

## Introduction

At the end of 2019, several cases of pneumonia with unknown etiology emerged in Wuhan, China, and on 7 January 2020, a novel coronavirus (2019-nCoV) was identified in the throat swab sample of one patient^1^. On 28 January 2020, the World Health Organization (WHO) declared 2019-nCoV infection a Public Health Emergency of International Concern and released interim guidelines on patient management^2^. On 11 February 2020, the International Committee on Taxonomy of Viruses renamed the virus as severe acute respiratory syndrome coronaviruse-2 (SARS-CoV-2), and WHO announced the epidemic disease caused by SARS-CoV-2 as coronavirus disease 2019 (COVID-19)^1^. As of the end of November 2020, COVID-19 has infected more than sixty million individuals and caused over 1.4 million deaths worldwide.

Population aging is among the largest problems in many countries. It is well known that older age is associated with a decline in immune competence^3^, increased comorbidities (e.g., hypertension, diabetes, coronary heart disease, and cerebrovascular disease), and greater risk of developing acute respiratory distress syndrome, likely because of a less rigorous immune response^4^. Thus, older adults face an elevated risk of infection with SARS-CoV-2. Increasing evidence conducted in many countries has shown that older adults are generally susceptible to SARS-CoV-2 and that there is a relatively high fatality rate among these populations^4–12^; therefore, older adults with COVID-19 should receive more attention. Unfortunately, the understanding of the clinical characteristics of COVID-19 and risk factors associated with death in older adult patients is limited. In this study, we collected clinical data on 281 older adult patients with severe COVID-19 in Wuhan, China, and further analysed their clinical characteristics and risk factors associated with their death to identify independent factors predicting all-cause mortality.

## Materials and methods

### Study design

This retrospective study was conducted in the intensive care units (ICUs) of the Infectious Disease Departments of Renmin Hospital of Wuhan University (Wuhan, China). Patients were diagnosed with COVID-19 according to the WHO’s interim guidance^13^. Clinical manifestations, results of computed tomography (CT) and real-time PCR for SARS-CoV-2 were included in the diagnostic criteria. All older patients with severe COVID-19 admitted from 30 January 2020 to 8 March 2020, were enrolled if they met at least one of the following three criteria: (1) respiratory distress with a respiratory rate of ≥ 30 breaths per minute; (2) oxygen saturation (fingertip pulse oximetry) of ≤ 93% in the resting state; or (3) arterial partial pressure of oxygen (PO_2_)/fraction of inspiration oxygen (FiO_2_) ≤ 300 mmHg (1 mmHg = 0.133 kPa), based on the recommendations of the National Institute for Viral Disease Control and Prevention, China^14^. The clinical outcome (mortality) was monitored up to 10 April 2020, the final follow-up date. No case was lost to follow-up for any reason. The present study was conducted in accordance with the Declaration of Helsinki (2013 edition) adopted by the World Medical Association^15^.The ethics committees of the First Affiliated Hospital of Dalian Medical University (PJ-KS-KY-2020–89) and the Renmin Hospital of Wuhan University (2020F033) approved this study and granted a waiver of informed consent. The study participants were divided into two age groups (60–79 years and ≥ 80 years) because of the particularly high risk of adverse health outcomes among those aged 80 years or older^16,17^.

### Data collection

The following types of data were extracted from electronic medical records using a data collection table: epidemiological data, demographic characteristics, medical history, contact history, signs and symptoms, comorbidities [hypertension, diabetes, coronary heart disease, cerebrovascular disease (cerebral haemorrhage, subarachnoid haemorrhage, ischemic stroke), chronic obstructive pulmonary disease, chronic kidney disease, chronic liver disease (chronic viral hepatitis, nonalcoholic fatty liver disease, alcoholic liver disease, primary and secondary cholestasis, liver cirrhosis), malignant tumors, thyroid diseases (hyperthyroidism, hypothyoidism, thyroiditis)], laboratory results [complete blood count, coagulation function, arterial blood gas, cellular immune, humoral immune, liver and renal functions, lactate dehydrogenase (LDH), electrolytes, osmotic pressure, lactic acid, C-reactive protein (CRP), myocardial markers, and procalcitonin (PCT)] on ICU admission, chest CT scans, time from onset to visit, time from onset to ICU admission, duration of SARS-CoV-2 positivity, and outcome. Clinical treatment measures (e.g., oxygen therapy, mechanical ventilation, kidney replacement therapy, antiviral therapy, antibiotics, glucocorticoid usage, traditional Chinese medicine, and nutritional support) were also collected. These treatments were based on the recommendations for COVID-19 diagnosis and treatment program (Fifth Edition) issued by the National Health Commission of the People’s Republic of China on 8 February 2020^14^. Sequential Organ Failure Assessment (SOFA) score was calculated on ICU admission based on age, medical history, vital signs, and laboratory results. All data were checked by two physicians (YJ and DC), and a third researcher (PG) adjudicated any differences in interpretation between the two primary reviewers.

### Laboratory procedures

To confirm SARS-CoV-2 infection, throat swab samples were obtained from all patients upon admission and tested using real-time reverse transcription polymerase chain reaction assays, following the same protocol described elsewhere^18^. Throat-swab samples were again obtained for SARS-CoV-2 polymerase chain reaction re-examination every other day after clinical remission of symptoms, including fever, cough, and dyspnea, but only qualitative data were available^14^. The criteria for discharge included absence of fever for at least 3 days, substantial improvement in both lungs in terms of clinical remission of respiratory symptoms and chest CT, and two negative SARS-CoV-2 RNA results from throat-swab samples obtained at least 24 h apart^14^.

### Definitions

Fever was defined as axillary temperature of over 37.3°C^5,6^. Acute liver injury was diagnosed if alanine aminotransferase or aspartate aminotransferase were over three times the upper limit of normal or total bilirubin was over two times the upper limit of normal^19^. Acute kidney injury was diagnosed according to the KDIGO clinical practice guidelines^20^. Acute respiratory distress syndrome was diagnosed according to the Berlin Definition^21^. Acute cardiac injury was diagnosed if serum levels of high-sensitivity troponin I were above the 99^th^ percentile upper reference limit or if new abnormalities were shown in electrocardiography or echocardiography^22^.

### Statistical analysis

We analysed the data using SPSS (Version 22.0) and presented them as medians and interquartile ranges (IQRs). We used the Pearson chi-squared test or Fisher’s exact test to compare demographic variables, signs and symptoms, comorbidities, treatment measures, and the 28-day all-cause mortality rate. We compared variables using the Mann–Whitney *U* test if a skewed distribution was confirmed using the Kolmogorov–Smirnov test. We used Spearman’s rank correlation coefficient to analyse correlations among the variables. We generated cumulative survival curves using the Kaplan–Meier method and compared them using the log-rank test. We conducted a binary logistic regression analysis to determine the risk factors associated with 28-day all-cause mortality, and reported the odds ratios and 95% confidence intervals. Additionally, we constructed receiver operating characteristic (ROC) curves and determined the areas under the ROC curve (AUCs). We compared AUCs using DeLong’s test. We also determined prognostic parameters, including sensitivity, specificity, positive predictive value, negative predictive value, Youden Index, positive likelihood ratio, and negative likelihood ratio, which was based on the optimal thresholds generated by analysing the ROC curves. Differences were considered significant if *P* < 0.05.

## Results

### Demographic and clinical characteristics

**Figure 1:**
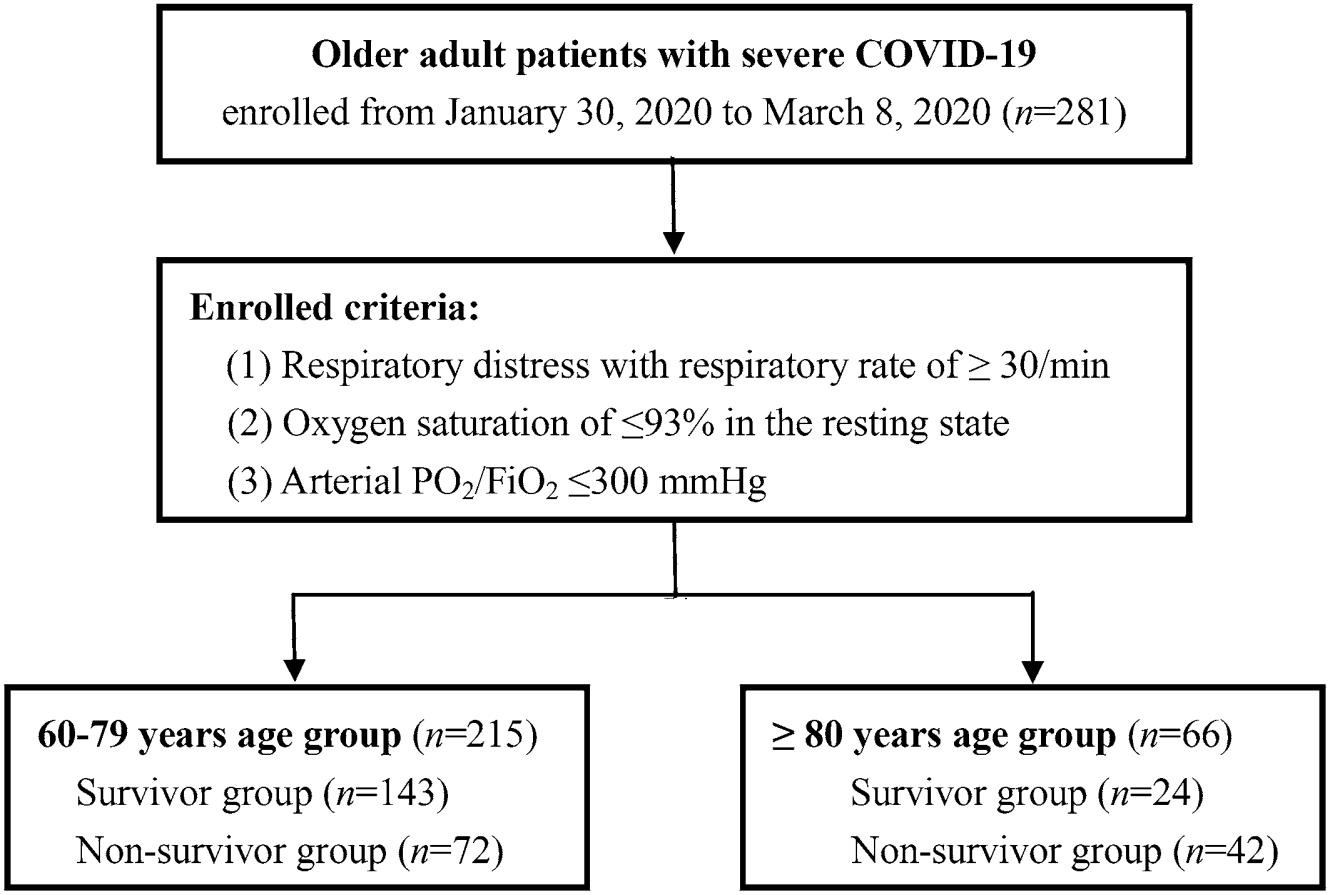
Flowchart of study participants. COVID-19, coronavirus disease 2019.

A total of 281 older adult patients with severe COVID-19 were included in the present study (Fig. 1). The median age was 70 years (IQR: 65–77 years), and age ranged from 60 to 95 years. Non-survivors had a significantly older age compared with survivors in either age group (Table 1). There were no significant sex differences between non-survivors and survivors in either age group.

**Table 1:** Baseline and clinical characteristics of the study cohort.

	60–79 years age group				≥ 80 years age group				*P* ^3^
	(*n* = 215)				(*n* = 66)				
	Total	Survivor	Non-survivor	*P* ^1^	Total	Survivor	Non-survivor	*P* ^2^	
		(*n* = 143)	(*n* = 72)			(*n* = 24)	(*n* = 42)		
Male [*n* (%)]	105 (48.8)	69 (48.3)	36 (50.0)	0.885	38 (57.6)	10 (41.7)	28 (66.7)	0.070	0.260
Age (years)	68 (64,72)	66 (63,71)	70 (67,74)	0.000	84 (81,85)	82 (81,85)	84 (82,87)	0.010	0.000
**Signs and symptoms [n (%)]**									
Fever	173 (80.5)	117 (81.8)	56 (77.8)	0.473	48 (72.7)	19 (79.2)	29 (69.0)	0.566	0.229
Dry cough	132 (61.4)	84 (58.7)	48 (66.7)	0.300	40 (60.6)	16 (66.7)	24 (57.1)	0.601	1.000
Fatigue	111 (51.6)	64 (44.8)	47 (65.3)	0.006	42 (63.6)	15 (62.5)	27 (64.3)	1.000	0.092
Dyspnea	82 (38.1)	52 (36.4)	30 (41.7)	0.461	31 (47.0)	9 (37.5)	22 (52.4)	0.309	0.251
Diarrhea	27 (12.6)	22 (15.4)	5 (6.9)	0.085	6 (9.1)	2 (8.3)	4 (9.5)	1.000	0.519
Nausea and vomitting	11 (5.1)	10 (7.0)	1 (1.4)	0.104	2 (3.0)	0 (0)	2 (4.8)	0.530	0.739
Runny nose	2 (0.9)	2 (1.4)	0 (0)	0.552	0 (0)	0 (0)	0 (0)	–	1.000
Sore throat	12 (5.6)	6 (4.2)	6 (8.3)	0.223	2 (3.0)	2 (8.3)	0 (0)	0.129	0.532
Anorexia	56 (26.0)	22 (15.4)	34 (47.2)	0.000	29 (43.9)	5 (20.8)	24 (57.1)	0.005	0.009
**Comorbidities [n (%)]**									
Hypertension	89 (41.4)	52 (36.4)	37 (51.4)	0.040	48 (72.7)	13 (54.2)	35 (83.3)	0.020	0.000
Diabetes	55 (25.6)	30 (21.0)	25 (34.7)	0.033	26 (39.4)	5 (20.8)	21 (50)	0.035	0.043
Coronary heart disease	43 (20.0)	27 (18.9)	16 (22.2)	0.590	27 (40.9)	11 (45.8)	16 (38.1)	0.608	0.001
Cerebrovascular disease	7 (3.3)	3 (2.1)	4 (5.6)	0.227	13 (19.7)	5 (20.8)	8 (19.0)	1.000	0.000
COPD	19 (8.8)	7 (4.9)	12 (16.7)	0.009	19 (28.8)	1 (4.2)	18 (42.9)	0.001	0.000
Chronic kidney disease	3 (1.4)	2 (1.4)	1 (1.4)	1.000	7 (10.6)	3 (12.5)	4 (9.5)	0.699	0.002
Chronic liver disease	9 (4.2)	6 (4.2)	3 (4.2)	1.000	0 (0)	0 (0)	0 (0)	–	0.122
Malignant tumors	14 (6.5)	7 (4.9)	7 (9.7)	0.240	0 (0)	0 (0)	0 (0)	–	0.046
Thyroid disease	1 (0.5)	1 (0.7)	0 (0)	1.000	4 (6.1)	2 (8.3)	2 (4.8)	0.618	0.012
**Treatment measures [n (%)]**									
Mechanical ventilation	37 (17.2)	4 (2.8)	33 (45.8)	0.000	14 (21.2)	0 (0)	14 (33.3)	1.000	0.468
High flow oxygen therapy	39 (18.1)	15 (10.5)	24 (33.3)	0.000	19 (28.8)	3 (12.5)	16 (38.1)	0.046	0.081
Methylprednisolone	114 (53.0)	73 (51.0)	41 (56.9)	0.470	34 (51.5)	12 (50.0)	22 (52.4)	1.000	0.888
Thymopentin injection	44 (20.5)	33 (23.1)	11 (15.3)	0.212	12 (18.2)	6 (25.0)	6 (14.3)	0.329	0.860
Gamma globulin injection	127 (59.1)	78 (54.5)	49 (68.1)	0.077	30 (45.5)	10 (41.7)	20 (47.6)	0.798	0.065
Antibiotics	172 (80.0)	103 (72.0)	69 (95.8)	0.000	61 (92.4)	21 (87.5)	40 (95.2)	0.345	0.024
Interferon-α injection	45 (20.9)	29 (20.3)	16 (22.2)	0.726	5 (7.6)	4 (16.7)	1 (2.4)	0.055	0.016
Oseltamivir	81 (37.7)	51 (35.7)	30 (41.7)	0.456	11 (16.7)	5 (20.8)	6 (14.3)	0.511	0.002
Abidor	154 (71.6)	102 (71.3)	52 (72.2)	1.000	43 (65.2)	15 (62.5)	28 (66.7)	0.792	0.357
Chloroquine/Hydroxychloroquine	34 (15.8)	27 (18.9)	7 (9.7)	0.112	8 (12.1)	5 (20.8)	3 (7.1)	0.128	0.556
Ribavirin	92 (42.8)	63 (44.1)	29 (40.3)	0.662	29 (43.9)	13 (54.2)	16 (38.1)	0.303	0.888
Kreiz	4 (1.9)	1 (0.7)	3 (4.2)	0.110	0 (0)	0 (0)	0 (0)	–	0.576
Lianhuaqingwen	134 (62.3)	88 (61.5)	46 (63.9)	0.767	25 (37.9)	11 (65.8)	14 (33.3)	0.429	0.001
Xuebijing injection	57 (26.5)	35 (24.5)	22 (30.6)	0.413	23 (34.8)	9 (37.5)	14 (33.3)	0.792	0.213
Parenteral nutrition	28 (13.0)	16 (11.2)	12 (16.7)	0.286	22 (33.3)	10 (41.7)	12 (28.6)	0.293	0.000
Enteral nutrition	24 (11.2)	11 (7.7)	13 (18.1)	0.037	21 (31.8)	5 (20.8)	16 (38.1)	0.178	0.000
Onset-visit time (days)	4.0 (1.0,7.0)	4.0 (1.0,7.0)	4.0 (1.0,6.0)	0.627	4.0 (1.0,7.0)	4.0 (1.0,8.0)	4.0 (1.0,7.0)	0.667	0.501
Onset-ICU time (days)	11.0 (8.0,15.0)	11.0 (8.0,16.0)	10.5 (9.0,14.8)	0.432	9.0 (6.0,12.0)	10.0 (7.0,12.0)	8.0 (4.8,12.3)	0.315	0.000
Duration of SARS-CoV-2 (days)	–	26.0 (19.0,34.0)	–	–	–	26.0 (22.3,36.8)	–	–	0.351
PO_2_/FiO_2_ (%)	175.8 (127.4,227.0)	202.5(163.0,266.3)	125.0 (91.0,180.1)	0.000	156.5 (117.8,241.5)	163.0 (125.3,290.0)	148.0 (102.4,241.5)	0.831	0.539
SOFA (score)	3 (2, 4)	2 (1, 3)	5 (3, 6)	0.000	4 (3, 6)	3 (3, 4)	5 (4, 6)	0.000	0.000
28-day mortality rate [*n* (%)]	72.0 (33.5)	–	–	–	42.0 (63.6)	–	–	–	0.000

Values are medians (interquartile ranges). *P*^1^ indicates the *P*-values for the comparison of survivors and non-survivors in the 60–79 years age group; *P*^2^ indicates the *P*-values for the comparison of survivors and non-survivors in the ≥ 80 years age group; *P*^3^ indicates the *P*-values for the comparison of the 60–79 years age group and the ≥ 80 years age group.

COPD, chronic obstructive pulmonary disease; ICU, intensive care unit; SOFA, Sequential Organ Function Assessment.

The most common symptoms at onset of illness in the 60–79 years age group (*n* = 215) and the ≥ 80 years age group (*n* = 66) were fever, dry cough, fatigue, and dyspnea, and there were no significant differences in the frequency of these conditions by age group. A higher percentage of patients in the ≥ 80 years age group had anorexia compared with those in the 60–79 years age group, and the percentage with anorexia was higher among non-survivors than among survivors in both age groups (*P* < 0.05, Table 1).

Over half of the older adult patients (69.4%, 195/281) had comorbidities, with hypertension being the most common, followed by diabetes, coronary heart disease, cerebrovascular disease, chronic obstructive pulmonary disease, and chronic kidney disease. Higher percentages of the patients in the ≥ 80 years age group had hypertension, coronary heart disease, cerebrovascular disease, and chronic kidney disease, compared with those in the 60–79 years age group (*P* < 0.05), with no significant differences between non-survivors and survivors. In addition, all the older adult patients had similar chest CT scan results—namely, bilateral ground-glass opacity or consolidation—on ICU admission.

### In-hospital treatments

Fifty-one (18.1%) older adult patients received mechanical ventilation (invasive or noninvasive), with no significant difference between the two age groups (Table 1). For patients receiving mechanical ventilation, only 5 patients (13.5%,5/37) survived in the 60–79 years age group, and 1 (7.1%,1/14) survived in the ≥ 80 years age group. Fifty-eight (20.6%) patients received high-flow oxygen therapy, and there was no significant difference between the two age groups; among these patients, the survival rate was 43.6% (17/39) in the 60–79 years age group and 20.1% (4/19) in the ≥ 80 years age group. These patients nearly all received antiviral treatments and traditional Chinese medicine such as Lianhuaqingwen (inhibiting the virus) and Xuebijing injection (antagonizing inflammation), with no significant differences between non-survivors and survivors or between the two age groups. Methylprednisolone (1–2 mg/kg, < 5 days), thymopentin injection, and gamma globulin injection were also commonly used to treat these patients, without significant differences by survivor status or age group. A total of 233 (82.9%) patients received antibiotics. Higher percentages of the patients in the ≥ 80 years age group received parenteral nutrition or enteral nutrition compared with those in the 60–79 years age group; there were no significant differences in these variables between non-survivors and survivors.

### Clinical course and outcomes

The median time from illness onset to visit and to ICU admission did not differ significantly between the two age groups (Table 1). For survivors, the duration from viral detection to negative SARS-CoV-2 result was not significantly different between the ≥ 80 years age group and the 60–79 years age group, and all non-survivors carried SARS-CoV-2 until death. PO_2_/FiO_2_ did not differ significantly between the two age groups, whereas the older patients who did not survive had a lower PO_2_/FiO_2_, compared with the survivors (Table 1).

**Figure 2:**
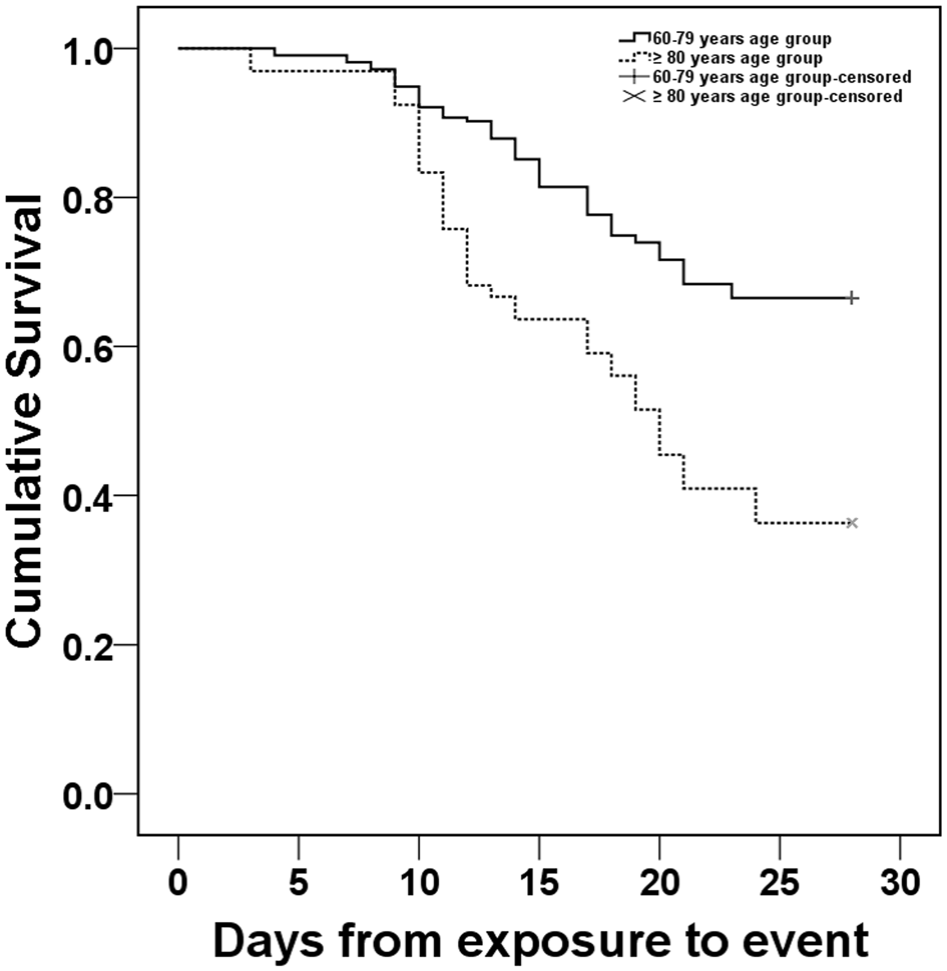
Kaplan–Meier survival curves for patients in the 60–79 years and the ≥ 80 years age groups. Patients in the ≥ 80 years age group had a significantly shorter median survival time compared with patients in the 60–79 years age group (20 days vs. 28.0 days); the log-rank test indicated a significant difference between the two survival curves (*P* < 0.001).

Higher SOFA scores were observed in the patients aged ≥ 80 years than in those aged 60–79 years, and non-survivors had higher SOFA scores than did survivors (Table 1). Likewise, the patients in the ≥ 80 years age group had higher all-cause mortality than did those in the 60–79 years age group (63.6% vs. 33.5%, *P* < 0.05). The patients in the ≥ 80 years age group had a significantly shorter median survival time, compared with the patients in the 60–79 years age group (20 days vs. 28.0 days, *P* < 0.05, Fig. 2).

### Comparison of laboratory findings

The comparisons of laboratory findings on ICU admission are summarized in Tables 2 and 3. Lymphocytopenia was commonly seen in older adult patients. The patients aged ≥ 80 years had a significantly lower lymphocyte percentage than did those aged 60–79 years, and non-survivors had a significantly lower lymphocyte percentage than did survivors. Non-survivors tended to have neutrophilia and a slightly elevated PCT, whereas neutrophils (both count and percentage) and PCT were nearly normal in survivors. In addition, among these patients, non-survivors had a significantly decreased platelet count, compared with non-survivors. These patients had significantly elevated D-dimer levels, and the patients aged ≥ 80 years had significantly higher D-dimer levels than did those aged 60–79 years. Furthermore, higher D-dimer levels were found in non-survivors than in survivors.

**Table 2:** Comparison of laboratory findings between the two age groups.

	Normal range	60–79 years age group (*n* = 215)	≥ 80 years age group (*n* = 66)	*P*
**Whole blood cell analysis**				
White blood cell (10^9^/L)	3.5–9.5	6.60 (4.53, 8.52)	7.63 (5.02, 11.01)	0.100
Neutrophil percentage (%)	40–75	76.5 (63.7, 88.1)	81.5 (68.7, 89.5)	0.057
Neutrophil count (× 10^9^/L)	1.8–6.3	4.86 (2.84, 7.12)	5.79 (3.90, 10.08)	0.031
Lymphocyte percentage (%)	20–50	15.8 (7.0, 25.0)	9.8 (6.6, 21.2)	0.022
Lymphocyte count (× 10^9^/L)	1.1–3.2	0.92 (0.62, 1.45)	0.87 (0.42, 1.33)	0.136
Monocyte count (× 10^9^/L)	0.1–0.6	0.40 (0.27, 0.58)	0.46 (0.30, 0.58)	0.438
Red blood cells (× 10^12^/L)	3.8–5.1	3.98 (3.57, 4.19)	3.90 (3.50, 4.34)	0.904
Hemoglobin (× g/L)	115–150	121.0 (109.0, 130.0)	120.5 (108.0, 136.3)	0.538
Platelets count (× 10^9^/L)	125–350	211.0 (159.0, 259.0)	176.0 (118.0, 241.0)	0.082
**Coagulation function parameters**				
Prothrombin time (second)	9–13	12.3 (11.7, 13.1)	12.7 (11.8, 14.2)	0.010
APTT (second)	25.0–31.3	27.50 (25.40, 31.10)	28.95 (26.60, 32.48)	0.051
Fibrinogen (g/L)	2–4	4.6 (3.2, 6.0)	4.3 (3.0, 5.8)	0.558
D-dimer (mg/L)	0–0.55	1.40 (0.64, 7.65)	4.58 (1.11, 22.43)	0.001
Antithrombin III activity (%)	80–120	83.8 (77.4, 91.3)	78.0 (68.0, 86.3)	0.001
**Cellular immune parameters**				
CD4+ percentage (%)	33–58	42.4 (34.8, 52.0)	38.8 (29.7, 44.8)	0.002
CD4+ count (/μL)	404–1612	350.0 (199.0, 547.0)	269.0 (177.5, 419.0)	0.009
CD8+ percentage (%)	13–39	18.0 (13.1, 25.0)	19.4 (12.3, 23.2)	0.471
CD8+ count (/μL)	220–1129	156.0 (73.0, 246.0)	108.0 (63.0, 236.0)	0.267
CD4+ /CD8+	0.9–2.0	2.19 (1.54, 3.63)	1.72 (1.06, 2.63)	0.016
CD19+ percentage (%)	13–39	17.7 (12.5, 24.3)	15.3 (11.1, 27.7)	0.447
CD19+ count (/μL)	80–616	123.0 (70.0, 209.0)	121.5 (65.0, 196.0)	0.649
CD16+ 56+ percentage (%)	6–26	13.5 (8.3, 21.8)	16.4 (12.8, 29.8)	0.057
CD16+ 56+ count (/μL)	84–724	112.0 (73.0, 180.0)	103.5 (60.0, 233.0)	0.875
**Liver injury markers**				
ALT (U/L)	9–50	24.0 (17.0, 40.0)	24.0 (15.8, 38.3)	0.743
AST (U/L)	15–40	30.0 (21.0, 45.0)	43.0 (26.8, 63.3)	0.003
Albumin (g/L)	40–55	34.3 (31.9, 37.8)	34.1 (31.6, 36.9)	0.258
Total bilirubin (μmol/L)	0–23	11.3 (8.4, 17.1)	11.7 (8.9, 19.2)	0.413
Lactate dehydrogenase (U/L)	120–250	318.0 (243.0, 477.0)	436.0 (263.0, 592.8)	0.003
**Kidney injury marker**				
Creatinine (μmol/L)	57–97	62.0 (50.0, 80.0)	94.0 (70.0, 120.0)	0.000
Urea (mmol/L)	2.6–7.5	5.99 (4.40,8.20)	11.00 (7.68,16.85)	0.000
Myocardial injury markers				
hs-TNI (ng/mL)	0–0.04	0.006 (0.006, 0.020)	0.061 (0.015, 0.490)	0.000
PRO-BNP (pg/mL)	0–1800	571.4 (164.4, 1438.0)	2347.9 (943.6, 4867.9)	0.000
Osmotic pressure (mosm/L)	280–310	286.0 (280.6, 294.1)	291.4 (282.8, 302.8)	0.005
Glucose (mmol/L)	3.9–6.1	6.31 (5.36,8.96)	5.92 (5.63,7.44)	0.644
K^+^ (mmol/L)	3.5–5.3	3.98 (3.53,4.37)	4.05 (3.72,4.58)	0.208
Na^+^ (mmol/L)	137–147	141 (138,145)	142 (137,145)	0.673
Lactic acid (mmol/L)	0.5–1.5	2.20 (1.70, 3.20)	2.20 (1.30, 3.43)	0.609
C-reactive protein (mg/L)	0–10	52.6 (15.5, 102.3)	91.4 (61.8, 165.2)	0.000
Procalcitonin (ng/mL)	0–0.1	0.07 (0.04, 0.18)	0.19 (0.08, 0.56)	0.000

Values are medians (interquartile ranges). *P* indicates the *P*-values for the comparison of the 60–79 years age group and the ≥ 80 years age group.

ALT, alanine aminotransferase; AST, aspartate aminotransferase; APTT, activated partial thromboplastin time; hs-TNI, high-sensitivity troponin I; PRO-BNP, precursor-B-type natriuretic peptide.

**Table 3:** Comparison of laboratory findings between survivors and non-survivors in the two age groups.

	60–79 years age group (*n* = 215)			≥ 80 years age group (*n* = 66)		
	Survivor (*n* = 143)	Non-survivor (*n* = 72)	*P* ^1^	Survivor (*n* = 24)	Non-survivor (*n* = 42)	*P* ^2^
**Whole blood cell analysis**						
White blood cell (10^9^/L)	5.44 (4.17, 7.20)	8.40 (6.63, 12.76)	0.000	5.62 (5.02, 8.55)	8.20 (5.32, 12.16)	0.088
Neutrophil percentage (%)	68.9 (58.2, 80.6)	90.0 (83.7, 93.2)	0.000	73.3 (66.7, 83.4)	85.3 (74.7, 91.7)	0.001
Neutrophil count (× 10^9^/L)	3.70 (2.41, 5.72)	7.42 (5.14, 11.85)	0.000	4.10 (3.49, 6.45)	6.62 (4.29, 10.76)	0.010
Lymphocyte percentage (%)	21.6 (14.3, 29.2)	6.3 (4.6, 10.6)	0.000	17.9 (10.2, 22.1)	9.0 (4.7, 16.5)	0.001
Lymphocyte count (× 10^9^/L)	1.01 (0.77, 1.58)	0.67 (0.39, 0.91)	0.000	1.11 (0.86, 1.43)	0.59 (0.36, 1.21)	0.009
Monocyte count (× 10^9^/L)	0.43 (0.29, 0.59)	0.37 (0.22, 0.57)	0.052	0.51 (0.33, 0.64)	0.41 (0.30, 0.55)	0.056
Red blood cells (× 10^12^/L)	3.98 (3.58, 4.20)	3.98 (3.43, 4.18)	0.490	3.64 (3.28, 3.95)	4.03 (3.61, 4.60)	0.002
Hemoglobin (× g/L)	122.0 (110.0, 130.0)	119.0 (107.0, 130.0)	0.193	110.0 (103.0, 120.0)	133.0 (118.5, 143.8)	0.000
Platelets count (× 10^9^/L)	215.0 (165.0, 260.0)	199.5 (132.0, 240.0)	0.010	218.5 (171.0, 266.0)	132.0 (90.3, 212.5)	0.001
**Coagulation function parameters**						
Prothrombin time (second)	12.0 (11.6, 12.7)	12.7 (12.0, 14.2)	0.000	13.3 (11.9, 14.1)	12.6 (11.8, 14.4)	0.779
APTT (second)	27.10 (24.80, 31.10)	28.50 (26.60, 31.45)	0.055	31.00 (25.38, 32.23)	28.80 (27.03, 32.40)	0.968
Fibrinogen (g/L)	4.6 (3.3, 5.9)	4.7 (2.8, 6.5)	0.760	4.2 (3.0, 5.3)	4.4 (2.9, 6.1)	0.292
D-dimer (mg/L)	0.85 (0.47, 1.83)	6.68 (2.08, 21.81)	0.000	1.23 (0.51, 18.07)	8.36 (2.47, 27.30)	0.004
Antithrombin III activity (%)	85.6 (79.4, 92.0)	79.7 (71.2, 88.2)	0.000	81.8 (69.4, 89.4)	77.5 (67.8, 82.3)	0.165
**Cellular immune parameters**						
CD4+ percentage (%)	43.4 (36.3, 51.5)	40.2 (31.7, 52.9)	0.079	39.3 (29.2, 42.3)	37.5 (29.8, 49.3)	0.594
CD4+ count (/μL)	468.0 (269.0, 650.0)	211.0 (123.0, 349.0)	0.000	402.0 (209.0, 457.0)	238.0 (137.3, 342.0)	0.072
CD8+ percentage (%)	20.3 (14.0, 27.4)	15.2 (11.5, 20.8)	0.003	22.4 (19.4, 25.3)	12.9 (9.7, 20.6)	0.000
CD8+ count (/μL)	200.0 (107.0, 292.0)	72.5 (39.3, 156.0)	0.000	222.0 (112.0, 303.8)	84.0 (50.3, 165.3)	0.000
CD4+ /CD8+	2.08 (1.52, 3.58)	2.61 (1.71, 3.74)	0.178	1.36 (1.06, 1.83)	2.22 (1.10, 4.58)	0.010
CD19+ percentage (%)	16.0 (11.2, 21.7)	23.4 (14.8, 31.8)	0.000	16.0 (13.5, 21.0)	13.6 (11.0, 31.1)	0.957
CD19+ count (/μL)	136.0 (92.0, 227.0)	89.0 (55.0, 162.0)	0.000	141.0 (105.3, 201.5)	105.0 (60.8, 197.8)	0.263
CD16+ 56+ percentage (%)	13.7 (8.6, 20.1)	12.7 (7.3, 26.9)	0.615	15.7 (13.0, 28.6)	16.4 (12.2, 29.8)	0.936
CD16+ 56+ count (/μL)	122.0 (85.0, 186.0)	79.0 (38.3, 146.0)	0.000	162.0 (69.0, 293.8)	94.0 (53.3, 169.8)	0.029
**Liver injury markers**						
ALT (U/L)	25.0 (17.0, 41.0)	23.0 (17.0, 35.0)	0.575	28.0 (15.3, 38.3)	23.0 (16.0, 38.3)	0.225
AST (U/L)	26.0 (18.0, 37.0)	35.0 (24.0, 63.0)	0.000	27.5 (17.0, 59.3)	43.0 (32.3, 70.5)	0.018
Albumin (g/L)	35.8 (32.9, 38.8)	31.7 (29.3, 34.1)	0.000	34.0 (31.8, 37.6)	34.4 (31.3, 36.4)	1.000
Total bilirubin (μmol/L)	10. 6 (8.3, 14.9)	15.0 (9.7, 23.9)	0.001	9.6 (7.8, 12.8)	15.1 (10.0, 23.8)	0.005
Lactate dehydrogenase (U/L)	272.0 (217.0, 332.0)	496.5 (401.8, 641.0)	0.000	263.0 (223.5, 379.5)	575.0 (409.0,668.5)	0.000
**Kidney injury marker**						
Creatinine (μmol/L)	59.0 (49.0, 75.0)	70.5 (51.3, 86.3)	0.110	79.0 (58.0, 106.0)	100.0 (77.0, 130.0)	0.067
Urea (mmol/L)	5.10 (3.90,6.80)	7.87 (6.03,10.40)	0.000	7.85 (5.10,10.59)	15.40 (10.30,21.05)	0.000
**Myocardial injury markers**						
hs-TNI (ng/mL)	0.006 (0.006, 0.010)	0.020 (0.006, 0.117)	0.000	0.015 (0.006, 0.084)	0.163 (0.045, 0.853)	0.000
PRO-BNP (pg/mL)	282.0 (91.6, 966.3)	866.9 (573.9,1558.2)	0.000	1572.1(681.9,4256.0)	3746.3(1087.4,5249.9)	0.182
Osmotic pressure (mosm/L)	285.2 (280.2, 290.6)	291.4 (281.5, 312.2)	0.001	283.0 (277.2, 286.4)	301.6 (293.7,309.6)	0.000
Glucose (mmol/L)	5.85 (5.17,8.65)	6.85 (5.86,10.01)	0.001	5.72 (5.41,5.85)	6.76 (5.74,9.21)	0.000
K^+^ (mmol/L)	3.96 (3.50,4.35)	4.05 (3.55,4.38)	0.768	3.94 (3.73,4.13)	4.14 (3.68,4.65)	0.095
Na^+^ (mmol/L)	141 (138,144)	142 (138,149)	0.124	139 (137,142)	144 (139,147)	0.000
Lactic acid (mmol/L)	2.00 (1.50, 2.80)	2.40 (1.80, 3.70)	0.002	1.55 (1.23, 2.70)	2.50 (1.53, 4.30)	0.051
C-reactive protein (mg/L)	36.3 (5.6, 83.7)	92.7 (55.2, 133.8)	0.000	64.1 (6.0, 90.9)	140.4 (83.6, 188.5)	0.000
Procalcitonin (ng/mL)	0.06 (0.04, 0.11)	0.18 (0.07, 0.42)	0.000	0.09 (0.04,0.23)	0.41 (0.15,1.58)	0.000

Values are medians (interquartile ranges). *P*^1^ indicates the *P*-values for the comparison of survivors and non-survivors in the 60–79 years age group; *P*^2^ indicates the *P*-values for the comparison of survivors and non-survivors in the ≥ 80 years age group.

ALT, alanine aminotransferase; AST, aspartate aminotransferase; APTT, activated partial thromboplastin time; hs-TNI, high-sensitivity troponin I; PRO-BNP, precursor-B-type natriuretic peptide.

The CD4+ and CD8+ counts and the CD4+ /CD8+ ratio were significantly lower than the normal range. Patients aged ≥ 80 years, especially those who did not survive, had a noticeably more compromised cellular immune response. This was indicated by a significantly lower CD4+ count and CD4+ /CD8+ ratio in the ≥ 80 years age group than in the 60–79 years age group. In addition, non-survivors aged ≥ 80 years had a significantly lower CD8+ count, and non-survivors aged 60–79 years had significantly lower CD4+ , CD8+ , CD19, and CD16+ 56 counts, compared with survivors.

For the two age groups, the median alanine aminotransferase, aspartate aminotransferase, and total bilirubin, as well as creatinine, were significantly elevated in the non-survivors, but these levels were within normal ranges in the survivors, indicating nearly unaffected liver and kidney function in surviving older adult patients. The median values of aspartate aminotransferase and creatinine in the non-survivors aged ≥ 80 years were higher than normal ranges, indicating slightly injured liver and kidney function in the patients aged ≥ 80 years who died (acute liver injury: 1.1%, 3/281 for survivors and 3.9%, 11/281 for non-survivors; acute kidney injury: 3.6%, 10/281 for survivors and 14.6%, 41/281 for non-survivors). LDH was significantly elevated in both age groups and was higher in the ≥ 80 years age group than in the 60–79 years age group and in non-survivors than in survivors.

Similarly, the median values for high-sensitivity troponin I and precursor-B-type natriuretic peptide were significantly elevated in the non-survivors in both age groups, but they were within normal ranges in the survivors, indicating nearly unaffected myocardial function in surviving older adult patients. The median values for high-sensitivity troponin I and precursor-B-type natriuretic peptide in the non-survivors aged ≥ 80 years were higher than normal values, indicating slightly injured myocardial function in non-surviving older adult patients aged ≥ 80 years (acute myocardial injury: 6.0%, 17/281 for survivors and 15.3%, 43/281 for non-survivors).

Median osmotic pressure was significantly higher in the ≥ 80 years age group than in the 60–79 years age group, with higher values seen in the non-survivors than in the survivors, although all values were within the normal range. However, patients with diabetes had significantly higher median osmotic pressure than did patients without diabetes in 60–79 years age group [291.9 (285.9, 312.5) vs. 284.3 (279.3, 291.2), *P* < 0.05]. There was no significant difference between patients with diabetes and patients without diabetes in ≥ 80 years age group [301.6 (283.9, 312.1) vs. 289.4 (282.8, 300.9),* P* = 0.109]. Median CRP was significantly elevated in both age groups, especially for those aged ≥ 80 years, and non-survivors had a higher value than did survivors. Median lactic acid was beyond the upper limit of normal in the two age groups, with a higher value in non-survivors than in survivors.

### Correlation of main laboratory findings with age and SOFA score

LDH, osmotic pressure, CRP, D-dimer, high-sensitivity troponin I, and PCT were positively correlated with older age and SOFA score (all *P* < 0.05, Table 4), whereas CD8+ and lymphocyte counts were negatively correlated with older age and SOFA score (both *P* < 0.05).

**Table 4:** Correlation of main laboratory findings with age and SOFA score in older adult patients with COVID-19.

	Age		SOFA	
	*r*	*P*	*r*	*P*
Age	–	–	0.312	0.000
SOFA	0.312	0.000	–	–
Lymphocyte count	− 0.088	0.143	− 0.151	0.011
CD8+ count	− 0.226	0.000	− 0.349	0.000
D-dimer	0.301	0.000	0.425	0.000
Lactate dehydrogenase	0.192	0.001	0.553	0.000
hs-TnI	0.480	0.000	0.486	0.000
Osmotic pressure	0.185	0.002	0.356	0.000
C-reactive protein	0.288	0.000	0.505	0.000
Procalcitonin	0.411	0.000	0.539	0.000

COVID-19, coronavirus disease 2019; hs-TnI, high-sensitivity troponin I; SOFA, Sequential Organ Failure Assessment.

### Risk factors associated with 28-day all-cause mortality

Considering the total sample size of our study (*n* = 281) and to avoid overfitting in the model, the variables were chosen for binary logistic regression analysis on the basis of previous findings and clinical constraints. We used 28-day all-cause mortality as the dependent variable and anorexia, comorbidities, CD8+ count, lymphocyte count, CRP, D-dimer, LDH, high-sensitivity troponin I, osmotic pressure, PCT, and SOFA score on ICU admission as the independent variables. Multicollinearity among the independent variables was checked using the variance inflation factor (VIF) because there was significant correlation between independent variables in the model^23^. We found that the VIF was less than 10 for each variable^23^. The binary logistic regression analysis showed that anorexia, comorbidities including hypertension, diabetes and COPD, LDH, osmotic pressure, and SOFA score were independent risk factors that were significantly associated with 28-day all-cause mortality in patients aged 60 years or older (Table 5).

**Table 5:** Risk factors associated with 28-day mortality in older adult patients with COVID-19.

	β value	Wald value	*P* value	OR value	95% CI
Anorexia	2.128	13.660	0.000	8.397	2.717–25.955
Hypertension	1.167	5.514	0.019	3.212	1.213–8.506
Diabetes	1.290	5.284	0.022	3.633	1.209–10.914
COPD	1.827	5.567	0.018	6.216	1.363–28.357
SOFA	0.562	8.293	0.004	1.754	1.197–2.571
Lymphocyte count	− 0.026	0.175	0.676	0.974	0.863–1.101
CD8+ count	− 0.003	1.515	0.218	0.997	0.992–1.002
D-dimer	0.013	1.786	0.181	1.013	0.994–1.033
Lactate dehydrogenase	0.006	9.091	0.003	1.006	1.002–1.010
hs-TnI	0.638	0.069	0.793	1.892	0.016–223.776
Osmotic pressure	0.039	3.894	0.048	1.040	1.000–1.081
C-reactive protein	0.007	2.380	0.123	1.007	0.998–1.016
Procalcitonin	0.120	0.325	0.569	1.128	0.746–1.706
Constant	− 18.467	9.996	0.002	0.000	

CI, confidence interval; COVID-19, coronavirus disease 2019; hs-TnI, high-sensitivity troponin I; OR, odds ratio; COPD, chronic obstructive pulmonary disease; SOFA, Sequential Organ Failure Assessment.

### Value of LDH, osmotic pressure and SOFA score for predicting 28-day all-cause mortality

**Figure 3:**
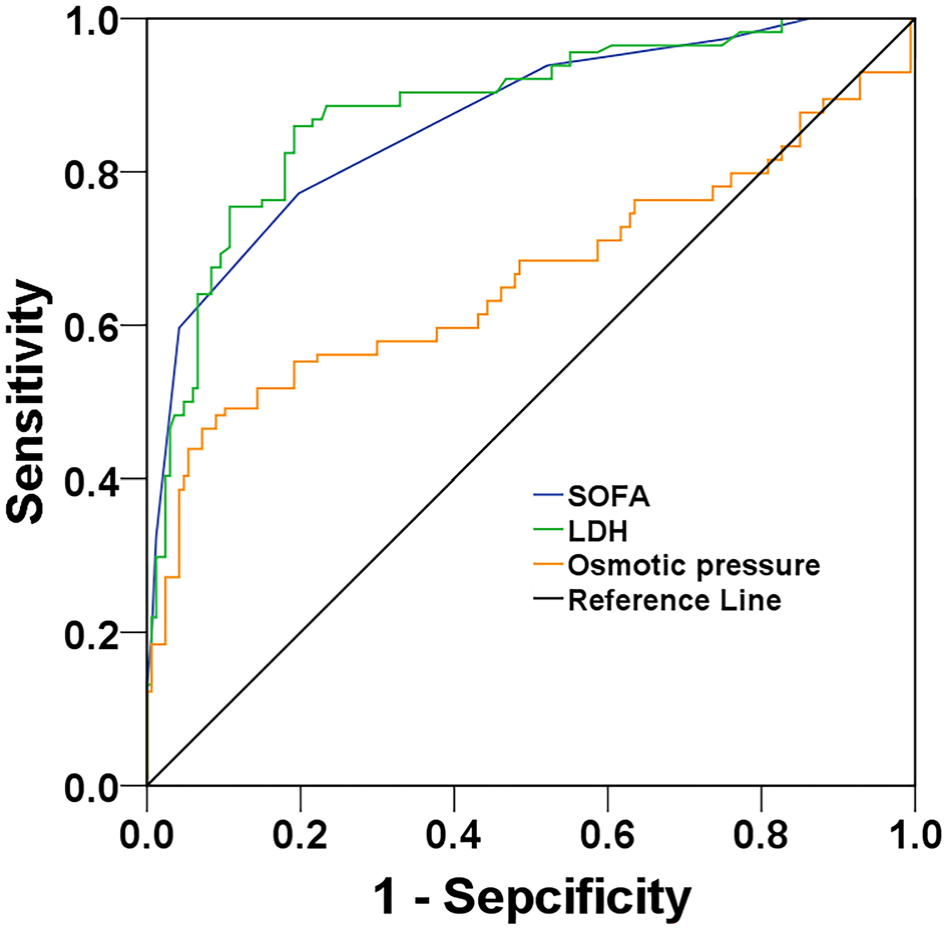
Receiver operating characteristic curves of age, LDH, osmotic pressure, and SOFA score for predicting 28-day mortality. LDH, lactate dehydrogenase; SOFA, sequential organ failure assessment.

LDH, osmotic pressure and SOFA score were valuable for predicting 28-day all-cause mortality (all *P* < 0.05, Fig. 3). LDH and SOFA score had larger AUCs than did osmotic pressure (Table 6). Noticeably, the AUC of LDH was larger than that of SOFA score, although there was no significant difference (*P* = 0.5744). Table 6 presents the performance of above variables in predicting 28-day mortality. Interestingly, the sensitivity (86.0% vs. 77.2%) and specificity (80.8% vs. 80.2%) of LDH were slightly superior to those of SOFA score.

**Table 6:** Performance of lactate dehydrogenase, osmotic pressure, and SOFA score on ICU admission in predicting 28-day all-cause mortality in older adult patients with COVID-19.

	AUC	*P* value	95% CI	Cutoff	Sensitivity (%)	Specificity (%)	PPV (%)	NPV (%)	Youden (%)	LR+	LR–
SOFA (score)	0.867	0.000	0.823–0.910	3.5	77.2	80.2	72.7	83.8	57.4	3.90	0.28
Lactate dehydrogenase (U/L)	0.882^a^	0.000	0.840–0.923	361.0	86.0	80.8	75.4	89.4	66.8	4.48	0.17
Osmotic pressure (mosm/L)	0.663^bc^	0.000	0.592–0.734	295.4	46.5	92.8	79.1	71.5	39.3	6.46	0.58

AUC, area under the receiver operating characteristic curve; CI, confidence interval; COVID-19, coronavirus disease 2019; ICU, intensive care unit; LR+ , positive likelihood ratio; LR–, negative likelihood ratio; NPV, negative predictive value; PPV, positive predictive value; SOFA, Sequential Organ Failure Assessment.

^a^*P* = 0.5744 (Z = 0.562) versus SOFA.

^b^*P* < 0.0001 (Z = 5.505) versus SOFA.

^c^*P* < 0.0001 (Z = 5.607) versus lactate dehydrogenase.

## Discussion

In the present study, we described the clinical characteristics of older adult patients with COVID-19, who have been reported to be at high risk of death^4–12^. We observed that the patients aged ≥ 80 years had higher all-cause mortality (63.6%) than did those aged 60–79 years (33.5%). Our findings for all-cause mortality in the two age groups were higher than the all-cause mortality reported in Beijing (18.8% among patients aged ≥ 80 years and 4.5% among those aged 60–79 years)^5^. This difference in results may be mainly because of different levels of COVID-19 severity among the older adult patients in the two studies. Renmin Hospital of Wuhan University was a designated hospital for severe cases of COVID-2019; therefore, the patients enrolled in the present study were nearly all classified as severe or critical cases, and the all-cause mortality rate may thus differ from those reported in other centers (especially outside Wuhan City). More importantly, the strength of the present study is the finding that, in addition to older age and comorbidities including hypertension, diabetes and COPD that have been observed in other studies^24–26^, anorexia, elevated plasma osmotic pressure and LDH, and high SOFA score also were independent factors associated with 28-day all-cause mortality in these older adult patients with severe COVID-19.

Consistent with previous reports^5,10^, the most common initial symptoms among our study participants were fever, dry cough, fatigue, and dyspnea. Noticeably, anorexia was also prevalent in these older adult patients. Furthermore, the patients in the ≥ 80 years age group had a higher percentage of anorexia than did those in the 60–79 years age group, and there was a higher percentage of anorexia in non-survivors than in survivors. This finding should not be neglected because anorexia is not only a clinical manifestation of COVID-19 but also a factor that may contribute to poor prognosis^27,28^. In the present study, we observed a significant association between anorexia and 28-day all-cause mortality in patients aged 60 years or older. Indeed, nutrition is an important element of health in the older adult population, and malnutrition caused by anorexia is associated with declines in immune function, functional status of vital organs, muscle function, cognitive function, and haemoglobin, as well as an increase in mortality^27^. Older adults often have reductions in appetite and energy expenditure, declines in biological and physiological functions, changes in cytokine and hormone levels, changes in fluid electrolyte regulation, delay in gastric emptying, and decreases in the senses of smell and taste^27,29^. Therefore, careful attention should be paid to nutritional status and nutritional support for older adult patients with COVID-19^28,30,31^, and oral supplements or enteral feeding should be considered for those at high-risk and for those unable to meet their daily nutritional requirements^27,31^.

Another interesting finding in this study was that elevated plasma osmotic pressure was positively related to age and SOFA score. Median osmotic pressure was significantly higher in the ≥ 80 years age group than in the 60–79 years age group, with a higher value in non-survivors than in survivors, although all median values were within the normal range. Elevated plasma osmotic pressure was also found to be significantly associated with 28-day all-cause mortality and was valuable for predicting 28-day all-cause mortality in patients aged 60 years or older, with a cutoff of 295.4 mosm/L. Elevated plasma osmotic pressure may be associated with stress hyperglycemia, electrolyte changes, less intake of water because of anorexia, and dehydration caused by fever. A particular range of osmolality of the body fluids is essential for the maintenance of cell volume^32^. Therefore, we recommend that plasma osmotic pressure should be measured at initial presentation and be continually monitored during hospitalization to enable timely and appropriate corrective action (e.g., appropriate increase of fluid infusion to decrease plasma osmotic pressure) if elevated plasma osmotic pressure is found.

In addition, consistent with previous reports^10,11^, we found LDH to be significantly elevated in the older adult patients and positively related to both age and SOFA score. Noticeably, LDH was the strongest predictor for 28-day all-cause mortality with a cutoff of 361.0 U/L, and had the best sensitivity and specificity. LDH is a group of cytoplasmic isoenzymes found especially in the liver, kidneys, striated muscle, and myocardium^33^. Hypoxia can induce LDH activity that reversibly catalyses the conversion of pyruvic acid to lactic acid without oxygen consumption in glucose metabolism, a process known as anaerobic glycolysis^33,34^. We speculated that severe hypoxia caused by lung injury, a predominant characteristic of patients with severe COVID-19, induces the increased activity and generation of LDH necessary for anaerobic glycolysis; this speculation was supported by the observation of elevated median lactic acid, a product of anaerobic glycolysis, in the older adult patients in our study. Plasma LDH was significantly elevated only when the cells of vital organs were injured in severe cases of COVID-19. Therefore, LDH might be considered a superior indicator reflecting severity and prognosis in patients with COVID-19.

We also observed that the older adult patients had significantly lower CD4+ and CD8+ T lymphocyte counts and a lower CD4+ /CD8+ ratio, compared with the normal ranges, which was consistent with the previous reports^35–38^. However, in the present study, cellular immune function was particularly compromised in the patients aged ≥ 80 years, especially those who did not survive, which was also supported by our observation that lymphocyte count (especially CD8+ count) was negatively correlated with age. T lymphocyte-mediated immunity is an adaptive process of developing antigen-specific T lymphocytes to protect against SARS-CoV-2 invasion. The T lymphocyte response under normal conditions is a finely balanced set of events regulated by the three subpopulations of reactive T cells (effector CD4+ , effector CD8+, and FoxP3+ CD4 or FoxP3+ CD8+ Tregs) and the associated cytokine storm^39^. CD4+ and CD8+ play a vital role in maintaining and regulating the stability of the internal immune environment^40^. The mechanism underlying the reduction of T cells may be associated with the direct invasion of SARS-CoV-2, which is similar to Middle East respiratory syndrome coronavirus infection^41^. In addition, the production of autoimmune antibodies induced by virus infection may cause growth inhibition and apoptosis of hematopoiesis, which can inhibit the production and differentiation of T cells^35,42^. Recent work in cellular immunology has also showed that viral infections make CD8+ T lymphocytes unable to sustain long-term activation and thus enter a stage of “exhaustion”^39,43^. Exhausted T lymphocytes are characterized by progressive loss of effector functions, high and sustained inhibitory receptor expression, metabolic dysregulation, poor memory, and homeostatic self-renewal^39^. Thus, it is plausible that a more compromised cellular immune response in the patients aged ≥ 80 years, especially among those who did not survive, may be caused by the exacerbation of immunosenescence with aging and T lymphocyte exhaustion caused by SARS-CoV-2 infection^3,44^. Accordingly, the restoration of T lymphocyte homeostasis from immunosenescence and from T cell exhaustion should be pivotal in the development of new and improved immuno-therapies for treating patients with COVID-19, and CD4+ and CD8+ T lymphocyte counts and CD4+ /CD8+ ratio may serve as indicators in evaluating the therapeutic effects of immuno-therapies. Unfortunately, decreased lymphocyte count was not associated with 28-day all-cause mortality in the present study, which was inconsistent with a previous study^45^. This discrepancy in findings may be explained by the fact that malnutrition and osmolality disturbance caused by anorexia and the decline of vital organ function with aging and multiple comorbidities more predominantly contribute to disease severity and death than do compromised cellular immune response in older adult patients with severe COVID-19.

The present study has several limitations. First, this was a retrospective study, which may limit the strength and reliability of our results. Second, we did not assess malnutrition or sarcopenia because this was a retrospective study, and thus no exact data on these items were available. Third, all enrolled patients were from a single center, which inevitably introduces selection bias. Fourth, immunoscenscence may be an important contributor to susceptibility and poor prognosis in older patients with severe viral infections such as SARS-CoV-2, cytomegalovirus (CMV), herpes simplex virus (HSV) and varicella zoster virus (VZV), which was not included in the present study. However, this topic will be investigated in the future. Fifth, differences in in-hospital treatments may have affected the prognosis of the older patients, despite the lack of significant differences for most treatments between the 60–79 years age group and the ≥ 80 years age group and between non-survivors and survivors. Sixth, we failed to rule out other potential bacterial or viral infections because tests for other viruses and bacteria were not performed on ICU admission. Finally, not all laboratory tests were conducted for all patients, so we were unable to analyse changes in inflammatory cytokines or their association with in-hospital mortality.

## Conclusions

Among older adult patients with severe COVID-19, those aged ≥ 80 years had higher all-cause mortality, compared with those aged 60–79 years. Anorexia, comorbidities including hypertension, diabetes and COPD, elevated plasma osmotic pressure and LDH, older age, and high SOFA score were independent factors associated with 28-day all-cause mortality in older adult patients with severe COVID-19. For these patients, LDH may have the highest superior predictive value for 28-day all-cause mortality in all examined factors.
